# Resolving the Complexity: A Comprehensive Review on Carbon Monoxide Diffusion Capacity in Chronic Obstructive Pulmonary Disease Patients

**DOI:** 10.7759/cureus.53492

**Published:** 2024-02-03

**Authors:** Lokesh Devalla, Babaji Ghewade, Ulhas Jadhav, Srinivasulareddy Annareddy

**Affiliations:** 1 Respiratory Medicine, Jawaharlal Nehru Medical College, Datta Meghe Institute of Higher Education and Research, Wardha, IND

**Keywords:** disease progression, respiratory physiology, pulmonary function tests, alveolar gas exchange, carbon monoxide (co) diffusion capacity, chronic obstructive pulmonary disease (copd)

## Abstract

This review comprehensively examines the intricate relationship between carbon monoxide (CO) diffusion capacity and chronic obstructive pulmonary disease (COPD). COPD, comprising chronic bronchitis and emphysema, presents a substantial global health burden, necessitating a nuanced understanding of pulmonary function parameters for effective diagnosis and management. The review delves into the physiological underpinnings, measurement techniques, and factors influencing CO diffusion capacity, emphasizing its pivotal role in assessing alveolar gas exchange efficiency. Key findings elucidate correlations between altered diffusion capacity and the severity of COPD, providing clinicians with valuable insights into disease progression. Considerations of age, gender, and ethnic disparities in normal CO diffusion capacity values highlight the importance of personalized interpretations. The clinical implications extend beyond diagnosis, influencing COPD management and prognostication, with potential applications in predicting treatment response. The review outlines avenues for future research, including advancements in measurement technologies and the exploration of novel imaging modalities and biomarkers. Ultimately, this review serves as a foundation for refining diagnostic approaches and treatment strategies and enhancing patient care in the dynamic landscape of COPD.

## Introduction and background

Chronic obstructive pulmonary disease (COPD) stands as a significant global health challenge, characterized by progressive airflow limitation and respiratory symptoms. This multifaceted respiratory condition encompasses chronic bronchitis, emphysema, and varying degrees of airway obstruction. With an escalating prevalence worldwide, COPD poses a substantial burden on healthcare systems and individual well-being [1]. It is a prevalent and complex respiratory disorder primarily associated with long-term exposure to harmful environmental factors, most notably cigarette smoke. It is characterized by persistent respiratory symptoms and a gradual decline in lung function, impacting the individual's ability to breathe effectively [2]. The spectrum of COPD includes chronic bronchitis, marked by inflammation of the airways, and emphysema, characterized by the destruction of lung tissue, leading to air sac enlargement and reduced elasticity [2].

Within the intricate landscape of COPD, assessing pulmonary function becomes paramount for diagnosis and management. One integral facet of this assessment is the measurement of carbon monoxide (CO) diffusion capacity, commonly evaluated through the diffusing capacity of the lungs for carbon monoxide (DLCO) test. Understanding CO diffusion capacity provides crucial insights into the efficiency of gas exchange within the alveoli, shedding light on the severity and progression of COPD [3]. This comprehensive review aims to dissect the intricate relationship between CO diffusion capacity and COPD. This review seeks to unravel the complexities surrounding CO diffusion capacity in individuals afflicted with COPD by scrutinizing existing literature, exploring the physiological underpinnings, and delving into measurement techniques. The scope of this review extends beyond the conventional boundaries of COPD literature, focusing specifically on the nuanced role of CO diffusion capacity in understanding the disease spectrum. By examining normal variations, alterations in COPD, and the clinical implications of CO diffusion capacity, this review aspires to provide a holistic perspective for clinicians, researchers, and healthcare practitioners. The significance lies in the potential for improved diagnostic accuracy, prognosis assessment, and the development of targeted therapeutic interventions in COPD management. As we embark on this exploration, we anticipate contributing to the broader dialogue on respiratory health and fostering advancements in understanding and treating COPD.

## Review

Measurement techniques for CO diffusion capacity

Pulmonary Function Tests

CO diffusion capacity assessment, also termed “transfer factor,” constitutes a crucial element within pulmonary function tests (PFTs) employed to evaluate lung function. Throughout this examination, the patient inhales a small quantity of CO, known for its heightened affinity to hemoglobin, and sustains breath retention briefly. The evaluation quantifies the transfer of CO from the lungs to the bloodstream, reflecting the lungs' efficacy in transferring gases [4,5]. Determinants of diffusion capacity encompass the pressure gradient and solubility of the gas, the ventilation-perfusion ratio of lung units, the rate of hemoglobin combination, and hemoglobin values [6]. CO, renowned for its affinity to hemoglobin and a parallel binding pathway to oxygen, is the predominant gas employed in this procedure [4].

The assessment of CO diffusion capacity yields pivotal insights into the gas exchange functionality of the alveolar-capillary unit in the lungs, enabling the diagnosis of diverse lung conditions and evaluation of treatment efficacy [5]. This assessment is a critical tool in appraising lung function, particularly pertinent in diagnosing and managing conditions such as COPD and emphysema [4]. Employing a single breath technique, this test, while informative, has faced criticism for measuring diffusion in a somewhat non-physiological context during maximal inhalation and breath retention, deviating from normal tidal volume breathing [6]. Novel methodologies, such as using nitric oxide (NO), are currently under exploration as potential alternatives, offering new avenues beyond the traditional single-breath technique [6]. The measurement of CO diffusion capacity emerges as an invaluable technique for assessing lung function, frequently employed in diagnosing and managing various respiratory conditions. It furnishes crucial insights into lung gas exchange functionality and assumes a substantial role in the comprehensive evaluation of pulmonary health.

Imaging Modalities

DLCO is a critical measurement assessing the lungs' capability to transfer CO from the alveoli to the bloodstream, mirroring the oxygen pathway. DLCO is quantified at full lung inflation during breath holding, representing total lung capacity (TLC) [5]. In this test, inhaled CO is employed due to its high hemoglobin affinity (measured in mL/min/mm Hg), signifying the volume of CO transferred per minute for each mm Hg of pressure difference across the total lung [7]. Various techniques are available for DLCO measurement, including the single-breath, steady-state, and rebreathing methods [8]. The single-breath technique, commonly used, involves inhaling a small CO amount and holding the breath for 10 seconds before exhalation [8], the steady-state technique requires inhaling a low CO concentration for extended periods, and the rebreathing technique involves re-inhaling a CO and gas mixture [7]. Although imaging modalities are typically not employed for DLCO measurement, given its nature as a functional test assessing gas exchange, innovative approaches such as hyperpolarized helium-3 MRI have been used to investigate the relationship between DLCO and lung function in COPD patients [3]. These methodologies contribute to a more comprehensive understanding of pulmonary function and its implications for respiratory conditions.

Normal CO diffusion capacity values and variations

Age-Related Changes

The evaluation of mean values for DLCO and DLCO per unit of alveolar ventilation (DLCO/VA) reveals gender-specific distinctions. In the male cohort, the average DLCO is documented at 28.05±5.07 mL/min/mmHg, accompanied by a DLCO/VA of 4.569±0.694 mL/min/mmHg/L. Conversely, females exhibit lower mean values, with DLCO at 20.79±4.03 mL/min/mmHg and DLCO/VA at 3.419±0.584 mL/min/mmHg/L [9].

Age-related shifts in DLCO are intricately intertwined with lung volume (TLC) and alveolar ventilation. The diffusion process across the alveolar-capillary interface is directly proportional to the alveolar surface area. A study by Stam et al. explored the influence of age on diffusion capacity in a cohort of 55 healthy individuals aged 70 years and older. The outcomes unveiled a discernible decline in DLCO with age, even after accounting for variations in alveolar volume. While the precise mechanism remains unconfirmed, the findings suggest potential modifications in the alveolar-capillary membrane [10].

Significant alterations in the carbon monoxide transfer factor (KCO) among adults as they age may be linked to changes in the microvasculature stemming from the loss of lung elasticity with advancing years [11]. The observed variations in normal CO diffusion capacity values between genders, coupled with age-related adjustments in DLCO and KCO, underscore dynamic shifts likely rooted in modifications of the alveolar-capillary membrane and the microvasculature. These nuanced relationships emphasize the importance of considering gender and age differentials when interpreting and applying DLCO and KCO values in clinical assessments.

Gender Differences

The anticipated normal values for DLCO in healthy nonsmoking adults highlight a mean DLCO of 28.05±5.07 mL/min/mmHg for men and 20.79±4.03 mL/min/mmHg for women. Interestingly, the DLCO/VA ratio showed no notable discrepancy between genders, indicating that while the alveolar ventilation corrected DLCO may not differ significantly between men and women [9], there is a discernible gender gap in DLCO values. Men tend to exhibit higher DLCO values compared to their female counterparts. Moreover, a study focusing on survivors of a non-specific condition revealed that among individuals with impaired diffusion capacity, there was no significant contrast between males and females concerning DLCO% predicted value and KCO% predicted value [12]. Furthermore, existing research underscores that women diagnosed with COPD generally manifest lower DLCO values despite having a higher forced expiratory volume in one second (FEV1) percent predicted value compared to men [13]. These collective findings strongly imply the existence of gender-based disparities in CO diffusion capacity, with women consistently displaying lower values than men. Such differences may be attributed to various factors, including distinctions in lung size and thoracic dimensions [14,15].

Ethnic and Racial Disparities

Average DLCO values exhibit variability influenced by age, sex, height, and race. A 2021 study highlighted the impact of race, revealing that it accounts for approximately 5-10% of the total variance in DLCO. After adjusting for sex, age, and height, the study found that blacks demonstrated a 3.9 mL/min/mmHg lower DLCO than whites [16]. Another study extended these observations, noting impairments in DLCO among significant race/ethnicity groups in spirometry, lung volumes, and DLCO at six months [17]. Notably, when examining individuals with impaired DLCO, DLCO% predicted value and KCO% predicted value did not show significant differences between males and females [12]. Additionally, a recent investigation into COPD revealed that women, despite having a higher FEV1 percent predicted value compared to men, exhibited lower DLCO values [13]. These findings underscore the nuanced impact of demographic factors on DLCO values and emphasize the importance of considering multiple variables in interpreting pulmonary function assessments.

Alterations in CO diffusion capacity in COPD

Mechanisms of Impaired Gas Exchange

The deviations observed in DLCO in COPD stem from the destruction of alveoli, resulting in a notable decrease in DLCO [3]. This impairment in DLCO is closely linked to heightened COPD symptoms, diminished exercise performance, and an elevated risk of severe exacerbations, even when accounting for other contributing factors [3]. Remarkably, DLCO emerges as the most robust predictor of lung function decline in a five-year follow-up study involving COPD patients [3,4]. Despite its significance, the acquisition of DLCO in patients remains inconsistent, and its additional benefit beyond more conventional tools remains to be determined [3]. Encouragingly, studies reveal that pulmonary rehabilitation (PR) can enhance DLCO in COPD patients, both with and without ventilation inhomogeneity [18]. Notably, in non-critical COVID-19 patients, evidence indicates an improvement in DLCO within the initial six months post-discharge [12]. These findings underscore the multifaceted nature of DLCO as an indicator in COPD, its potential responsiveness to interventions such as PR, and its relevance in respiratory recovery, even in non-COPD respiratory conditions such as post-COVID-19 recovery.

Relationship Between CO Diffusion Capacity and Disease Severity

The association between DLCO and disease severity has been investigated in various respiratory conditions, notably COPD and pulmonary hypertension (PH). Within the context of COPD, a compromised DLCO has demonstrated connections with heightened COPD symptoms, diminished exercise performance, and an increased risk of severe exacerbations, even when considering other influencing factors [3,4]. Notably, in COPD patients with emphysema, characterized by alveolar destruction, a decrease in DLCO is observed, typically accompanied by an average or slightly reduced carbon monoxide transfer factor (KCO) [7].

In the realm of PH, DLCO has emerged as a noteworthy factor negatively associated with patient survival. Various factors, encompassing reduction in alveoli-capillary pulmonary micro-vessel area, changes in alveolar capillary volume, alterations in lung perfusion, smoking habits, and the presence of potential intrapulmonary or intracardiac shunts, contribute to the observed modifications in DLCO [19]. These collective findings underscore the significance of DLCO as a valuable marker for disease severity in respiratory conditions such as COPD and PH. The impairment in DLCO aligns with worse clinical outcomes and establishes itself as a crucial parameter in the comprehensive assessment and management of these respiratory diseases.

Correlation With Other Pulmonary Function Parameters

Adolescent idiopathic scoliosis: A comprehensive study on adolescent idiopathic scoliosis (AIS) delved into the intricate relationship between radiological parameters and lung function. Notably, parameters such as MT-Cobb (Cobb angle in scoliosis measurement), MT-AVB-R (atrioventricular block risk), MT-AVT (atrioventricular transmission), and MT-RH (respiratory rate and heart rate) negatively correlated with pulmonary function [20]. This correlation suggests that thoracic and spinal deformities in AIS may exert a discernible impact on the individual's pulmonary function, emphasizing the need for a holistic understanding of the physiological implications of scoliotic conditions.

Idiopathic interstitial pneumonia: Within the realm of idiopathic interstitial pneumonia (IIP), a meticulous exploration using high-resolution computed tomography (HRCT) showcased a substantial correlation with PFTs. Importantly, forced vital capacity (FVC) emerged as a more robust correlate compared to TLC [21]. This observation underscores the potential utility of HRCT in comprehensively assessing pulmonary function in patients with IIP, providing valuable insights for clinicians in the diagnostic and therapeutic realms.

General population: A cross-sectional study conducted in the general population sheds light on the intricate interplay between body composition factors and pulmonary function. Factors such as total body water, protein content, mineral content, fat-free mass, and skeletal muscle mass exhibited positive correlations with FVC and FEV1 [22]. This discerned relationship underscores the influence of body composition on pulmonary function, offering valuable insights into the broader determinants of respiratory health within the general populace.

Pulmonary hypertension: Within the domain of PH, a study focusing on patients with idiopathic PH unearthed a significant negative association between DLCO and patient survival [19]. This compelling finding underscores the potential of DLCO as a marker of severity in PH, emphasizing its clinical relevance in prognostication and possibly guiding therapeutic interventions for better patient outcomes.

Clinical implications and diagnostic utility

Use of CO Diffusion Capacity in Diagnosing COPD

The assessment of DLCO stands as a valuable tool in evaluating COPD. Multiple studies have demonstrated that a decline in DLCO is linked to heightened COPD symptoms, diminished exercise performance, and an increased risk of severe exacerbations. Importantly, these associations persist even after considering standard diagnostic tools such as spirometry and CT imaging [3]. This suggests that DLCO testing contributes additional diagnostic utility beyond the more frequently employed assessment methods. The independent correlation between DLCO and COPD morbidity implies that a lower DLCO may indicate underlying disease severity [3]. Consequently, integrating DLCO testing into the diagnostic workup for COPD proves valuable, offering insights not captured by other tests. This becomes particularly crucial for early detection and effective management of COPD, ultimately leading to improved treatment outcomes and patient care [3]. The use of DLCO in COPD diagnosis is substantiated by evidence showcasing its association with heightened COPD symptoms, compromised exercise performance, and an elevated risk of severe exacerbations. This underscores the diagnostic efficacy of DLCO testing, positioning it as a crucial component in the comprehensive assessment of COPD.

Prognostic Value in COPD Progression

The prognostic significance of DLCO emerges prominently in the context of COPD progression. Impairment in DLCO is intricately linked to an array of adverse outcomes in COPD patients, encompassing heightened symptoms, compromised exercise performance, and an elevated risk of severe exacerbations, even when accounting for other contributing factors [3]. A notable study further underscored the prognostic value of DLCO by revealing that for every 10% predicted decrease in DLCO, there was a concomitant exacerbation of symptoms, a decline in overall quality of life, compromised exercise capacity, and an increased incidence of severe exacerbations among COPD patients [3]. These compelling findings collectively affirm the pivotal role of DLCO as a prognostic indicator, providing valuable insights into the trajectory of COPD progression and the associated morbidity. Integrating DLCO assessments into COPD prognostication enhances our ability to predict disease outcomes and holds promise for tailoring interventions to optimize patient care and improve long-term management strategies.

Monitoring Treatment Response

Vigilance over treatment response is a critical facet in the management of diverse diseases, such as COPD. Employing various methods such as regular history taking, physical examination, chest radiographs, and laboratory monitoring becomes integral in this process [23,24]. Specifically in COPD, the assessment of treatment response involves tracking changes in lung function parameters such as FEV1 and DLCO [25]. Additionally, monitoring extends to changes in symptoms, exercise performance, and exacerbation risk, providing a comprehensive evaluation of treatment efficacy in COPD patients [25]. In different medical contexts, such as breast cancer and multiple myeloma, monitoring treatment response takes on a distinct character. Here, assessing changes in measurable residual disease and circulating tumor DNA serves as crucial indicators of treatment effectiveness [26,27]. The meticulous monitoring of treatment response emerges as a pivotal strategy in managing diverse diseases. The choice of monitoring methods varies depending on the disease, emphasizing the importance of tailored approaches to ensure optimal patient care and therapeutic outcomes.

Challenges and limitations

Variability in Measurement Techniques

A significant challenge and limitation in measuring DLCO lies in the inherent variability across measurement techniques [28]. The conventional single-breath measurement of CO uptake assumes instantaneous lung filling, overlooking that inspiration and expiration demand several seconds. The dynamic changes in gas volume during these periods must be duly considered in the ensuing calculations [28]. Addressing this challenge, Ogilvie et al. introduced a standardized clinical method for determining DLCO in 1957, incorporating a tracer [28]. Among the various methods, the approach proposed by Jones and Meade is particularly recommended for standardization purposes. This method possesses theoretical merit, as it empirically accounts for the effects of inspiratory and expiratory gas flows [28]. Alternatively, measuring the exhaled gas from the subject before inhaling the test gas offers an optional calculation method [28]. A crucial requirement in this process is the need for gas analyzer drift to be ≤10 ppm over 30 seconds for CO and ≤0.5% of full scale over the same duration [28]. Despite these methodological challenges, DLCO remains a vital parameter for assessing lung function in COPD patients. The association between impaired DLCO and heightened COPD symptoms, compromised exercise performance, and an increased risk of severe exacerbations underscores its clinical relevance [3]. As research and standardization efforts continue, the refinement of measurement techniques holds promise in enhancing the accuracy and reliability of DLCO assessments in evaluating pulmonary function.

Interpretation Challenges

The comprehension of the challenges associated with PR for patients with COPD necessitates an exploration of the diverse barriers that impede its effectiveness. A qualitative study has shed light on several impediments to PR for COPD patients, revealing factors such as poor coping with COPD, limited access to PR services, elevated levels of anxiety, insufficient insurance coverage for PR services, ineffective PR planning, and disruptions in continuity of care [29]. These barriers span across personal, familial, social, financial, organizational, and governmental realms, underscoring the intricate nature of the PR process for individuals grappling with COPD. In tandem, the World Health Organization (WHO) underscores the pivotal role of PR in the overall management of COPD. While COPD remains incurable, PR serves as an indispensable component of its treatment, aiming to enhance the quality of life and functional capacity of those afflicted [30]. However, the study's revelations emphasize the need for targeted strategies to overcome the identified barriers to amplify the effectiveness of PR for COPD patients. The challenges in interpreting the effectiveness of PR for COPD patients revolve around recognizing and addressing the multifaceted barriers that influence its implementation and outcomes. These barriers, spanning from individual to systemic levels, mandate comprehensive mitigation strategies to enhance PR delivery for individuals contending with COPD.

Confounding Factors

Smoking: Smoking stands as a primary causative factor for COPD and simultaneously exerts an influence on serum soluble receptor for advanced glycation end products (sRAGE) levels. This dual impact introduces significant variability in serum sRAGE levels across individuals [31]. Notably, adopting smoking cessation measures in the hours preceding blood sampling is suggested to mitigate the interindividual variations in serum sRAGE levels, emphasizing the dynamic relationship between smoking behavior and biomarker levels [31].

COPD exacerbations: During COPD exacerbations, there is a noteworthy decrease in serum sRAGE levels. Intriguingly, this decrease occurs without a concurrent difference in the gene expression encoding RAGE in granulocytes [31]. While this observation highlights a potential link between exacerbations and altered sRAGE levels, further investigations are warranted to comprehensively understand the impact of COPD exacerbations on serum sRAGE levels [31].

Genetic factors: Beyond conventional risk factors, untraditional contributors to COPD encompass genetic factors, prolonged asthma, exposure to outdoor air pollution, and secondhand smoke. Collectively, these elements contribute to the intricate web of influences that shape the development and progression of COPD [32].

Low body mass index: Low body mass index (BMI) indicates poor nutritional status, but, paradoxically, individuals in the lower tertile of BMI may face an elevated risk of developing COPD [33]. This apparent contradiction emphasizes the nuanced relationship between BMI and COPD risk, underlining the importance of considering BMI within a broader context of individual health factors.

Weight loss: Weight loss emerges as a potential confounding factor in assessing COPD, mainly as it can be induced by both COPD and lung cancer. Navigating the intricacies of weight loss as a confounder becomes crucial in accurately interpreting clinical data and outcomes in individuals with COPD [34].

Future directions and research opportunities

Advancements in Measurement Technologies

Machine accuracy measurement: Advancements in measurement and simulation techniques have propelled micro-measurement accuracy to a remarkable nano-scale level. This progress includes using sophisticated tools such as 3D fiber probes for radial probing and implementing non-contact autofocus measurement systems [35]. These cutting-edge methods enhance precision in measurement and extend the capabilities of micro-level accuracy, opening new possibilities for applications in various scientific and industrial domains.

Remote measurement technology for e-healthcare: The landscape of remote monitoring technologies has undergone a transformative shift, integrating wireless signal processing, artificial intelligence, and advanced algorithms for analyzing patient health data. This evolution can revolutionize healthcare practices, improving patient outcomes by detecting vital signs, wearable physiological monitoring, and health monitoring facilitated by biomedical radar applications [36]. This intersection of technology and healthcare can enhance preventive and proactive healthcare strategies.

Quantum sensing: Quantum sensing is a groundbreaking frontier in measurement and detection technology, introducing unprecedented levels of precision and accuracy. These compact and portable devices find applications across diverse fields, including medicine, telecommunications, and environmental monitoring. Quantum sensing delivers precise measurements and exhibits immunity to external disturbances, presenting limitless possibilities for the future of measurement and detection technology [37]. Its impact extends beyond conventional boundaries, offering novel solutions in various scientific and industrial domains.

Field instrumentation: The current trajectory in the field of instrumentation focuses on smart manufacturing, digital instrumentation, advanced analytics, and the industrial internet of things (IIoT). This entails leveraging wireless sensor networks, connectivity technology, and industrial connections to enhance operational capabilities and productivity [38]. Integrating these technologies heralds a new era in instrumentation, fostering improved efficiency and responsiveness in industrial processes.

Oil flow measurement technologies: Significant strides have been made in oil flow measurement technologies, crucial for accurately quantifying volumes of oil and gas. The ongoing evolution in fluid flow measurement technologies carries profound implications for the oil and gas industry [39]. These advancements enhance the accuracy and reliability of measurements, contributing to more efficient and sustainable practices in the extraction and use of oil and gas resources.

Potential Biomarkers for CO Diffusion Capacity

Several biomarkers have demonstrated associations with altered lung diffusion, shedding light on potential indicators of impaired diffusing capacity for carbon monoxide (DLCO). In a study, patients exhibiting modified lung diffusion presented elevated levels of matrix metalloproteinase-7 (MMP-7) compared to those with regular diffusion (11.54±8.96 vs 6.71±4.25, p=0.001) [40]. Similarly, the biomarker periostin displayed higher levels in individuals with altered lung diffusion (1.11±0.07 vs 0.84±0.40, p=0.001) [40]. Additionally, the peak value of D-dimer, a crucial clinical parameter for patients requiring respiratory support, showed a significant association with DLCO<80% of predicted values at six months (p=0.056) [40]. Gender differences also emerged as a factor, with female sex being linked to diffusion impairment at six months (OR: 2.97, 95% CI 1.74-5.06, p=0.001) [40]. Furthermore, age was associated with diffusion impairment at six months (OR: 1.03, 95% CI: 1.01-1.05, p=0.005), signifying age as a potential influencing factor in DLCO alterations [40]. The peak respiratory acute lung injury (RALE) score, a clinical scoring system, was also identified as a predictor, with higher scores correlating with diffusion impairment at six months (OR: 1.22, 95% CI 1.06-1.40, p=0.005) [40]. These findings underscore the potential utility of MMP-7, periostin, D-dimer, gender, age, and peak RALE score as predictors or indicators of DLCO impairment. Further investigations into these biomarkers across diverse patient populations hold promise for refining our understanding of their predictive value and enhancing their clinical applicability in assessing lung function.

Innovative Therapeutic Approaches

In COPD treatment, several innovative approaches are being explored to enhance patient outcomes and provide more targeted care. One avenue of exploration is stem cell therapy, a treatment method involving the injection of stem cells into the body to facilitate the regeneration of damaged lung tissue. Ongoing research endeavors seek to unravel this novel approach's potential in harnessing stem cells' regenerative capabilities to improve lung function in COPD patients [41]. Another promising frontier is gene therapy, which holds significant potential in addressing COPD by targeting the underlying genetic factors contributing to the disease. Through the manipulation or modification of specific genes, this therapeutic avenue aims to address the root causes of COPD, offering the prospect of more personalized and targeted treatment options for individuals dealing with this chronic respiratory condition [42].

The exploration of monoclonal antibody treatment represents a notable development in COPD care. Targeted monoclonal antibody treatments, including mepolizumab, reslizumab, benralizumab, and dupilumab, have improved asthma symptoms. Researchers are now investigating the potential of these targeted treatments in reducing symptoms and enhancing overall well-being in COPD patients, ushering in a new era of precision medicine for respiratory conditions [43]. PR is a comprehensive program designed to address various facets of COPD management. Focused on improving physical conditioning, teaching effective breathing techniques, and enhancing overall well-being, this holistic approach empowers COPD patients with valuable tools and strategies for better symptom management and improved quality of life [43].

With technological advancements, smart spirals and inhalers are emerging as transformative tools. These devices, capable of connecting to smartphones, enable the tracking and analysis of breathing patterns. This real-time data offers the potential for more personalized management approaches by providing insights into lung function and respiratory health [41]. Telemedicine and remote care represent critical solutions to improve access to healthcare services for COPD patients, particularly those with limited mobility or residing in remote areas. By facilitating remote monitoring, consultations, and support, these technologies play a crucial role in enhancing the overall management of COPD and ensuring that patients receive timely and effective care [41]. The exploration of lung restoration Therapies goes beyond stem cell interventions. This includes innovative approaches like smartwatches to monitor lung function and detect changes. These strategies aim to optimize symptom management, enhance the quality of life, and ultimately revolutionize outcomes for individuals grappling with the challenges of COPD [41]. As research and clinical trials continue, these diverse and promising approaches mark a transformative phase in COPD management, offering new avenues for treatment and personalized care strategies.

## Conclusions

In conclusion, this review illuminates the critical role of CO diffusion capacity in the landscape of COPD. Through an exploration of key findings, the review underscores the significance of CO diffusion capacity as a pivotal parameter for assessing alveolar gas exchange efficiency. The correlation between altered diffusion capacity and COPD severity emerges as a central theme, offering clinicians nuanced insights into disease progression. Moreover, considerations of age, gender, and ethnic disparities in normal CO diffusion capacity values provide a foundation for personalized interpretations across diverse populations. The interconnected nature of CO diffusion capacity with other pulmonary function parameters further accentuates its clinical relevance. The clinical implications of these findings extend beyond diagnostic applications, shaping COPD management and prognostication. CO diffusion capacity assessment facilitates early detection and accurate staging of COPD and holds promise in predicting treatment response and overall patient outcomes. This calls for a more tailored and precise approach to therapeutic interventions. The review suggests avenues for future research, including advancements in measurement technologies and the exploration of novel imaging modalities and biomarkers. Additionally, investigating the impact of emerging therapeutic strategies on diffusion capacity could pave the way for innovative interventions to improve gas exchange efficiency and mitigate disease progression. This comprehensive review serves as a foundation for refining diagnostic approaches and treatment strategies and ultimately enhancing patient care in COPD.
